# Editorial: Molecular Mechanisms in Stress and Trauma Related Disorders

**DOI:** 10.3389/fpsyt.2020.00103

**Published:** 2020-03-03

**Authors:** Anthony S. Zannas, Murray B. Stein, George P. Chrousos

**Affiliations:** ^1^ Department of Psychiatry, University of North Carolina, Chapel Hill, NC, United States; ^2^ Department of Genetics, University of North Carolina, Chapel Hill, NC, United States; ^3^ Department of Psychiatry and Behavioral Sciences, Duke University Medical Center, Durham, NC, United States; ^4^ Institute for Trauma Recovery, University of North Carolina School of Medicine, Chapel Hill, NC, United States; ^5^ Department of Family Medicine & Public Health, University of California, San Diego, La Jolla, CA, United States; ^6^ Department of Psychiatry, University of California, San Diego, La Jolla, CA, United States; ^7^ University Research Institute of Maternal and Child Health & Precision Medicine, National and Kapodistrian University of Athens, Athens, Greece; ^8^ Center for Adolescent Medicine and UNESCO Chair on Adolescent Health Care, First Department of Pediatrics, School of Medicine, National and Kapodistrian University of Athens, Aghia Sophia Children's Hospital, Athens, Greece

**Keywords:** autophagy, circadian biology, epigenetics, inflammation, major depression, posttraumatic stress disorder, psychosocial stress

Psychosocial stress is ubiquitous in modern societies and represents a potentially preventable risk factor for a host of aberrant phenotypes (1). Interestingly, despite their ubiquity, psychosocial stressors can elicit diverse and often unpredictable psychologic and somatic outcomes among exposed individuals. What constellation of mechanisms determines whether some individuals will suffer a variety of negative consequences in the face of adverse environments, whereas others will remain intact or even thrive during periods of stress exposure? A body of evidence has suggested that this interindividual variability may result from complex molecular mechanisms that collectively shape individual responses and outcomes to stress at the cellular, physiological, neuroendocrine, and behavioral levels. However, the exact molecular contributors to such responses and outcomes are poorly understood. In this Research Topic, we present a series of both original studies and review articles that shed new light into how stress-related conditions may be influenced by processes such as inflammation, circadian biology, intracellular signaling pathways, oxidative stress, and epigenetics.

Stress exposure can trigger episodes of major depression (2). Furthermore, both MDD and childhood maltreatment have been previously shown to promote inflammation in a potentially synergistic manner (3), yet the underlying mechanisms are still incompletely understood. In an original study, de Punder et al. demonstrate that only patients with both depression and a history of childhood adversity exhibit heightened inflammation, whereas depressed subjects without childhood adversity have inflammatory profiles similar to those of control subjects. In a systematic review and meta-analysis, Perrin et al. show that increased inflammation in depression is associated with glucocorticoid resistance, primarily indicated by higher levels and impaired suppression of the stress hormone cortisol. Together these findings suggest the existence of inflammatory subtypes of major depression that may be characterized by previous exposure to childhood adversity and/or aberrant glucocorticoid signaling.

Another key molecular process addressed herein is circadian biology and its particular involvement in trauma-related disorders. In an original study, Linnstaedt et al. show that genetic polymorphisms in circadian pathway genes influence risk for the development of posttraumatic stress symptoms following exposure to multiple types of psychologic trauma, including motor vehicle accidents, sexual assault, and burn injury. Complementarily, Agorastos et al. review how the stress system interacts at multiple levels with circadian biology and the potential relevance of these interactions for posttraumatic stress disorder. These articles underscore the importance of further dissecting the role of circadian biology in stress and trauma-related disorders.

Three original articles address how chemical modifications of nucleic acids, acting at multiple levels to influence DNA and RNA integrity and function, may be implicated in stress-related disorders. He et al. examine DNA methylation—one of the critical epigenetic mechanisms that regulate gene expression—and show that childhood maltreatment in humans is associated with higher, whereas bipolar disorder with lower, blood methylation levels of the gene expressing the cytokine and stem cell factor KIT ligand. Dick et al. employ a mouse model of chronic social defeat stress and show that chronic stress can set into motion adenosine-to-inosine RNA editing, a co-/posttranscriptional RNA modification that influences protein isoform expression, within the corticolimbic regions of the mouse brain). Boeck et al. examine oxidative stress and DNA damage in *postpartum* women and show that exposure to childhood maltreatment is positively associated with levels of 8-isoprostane, a marker of lipid peroxidation, but not with markers of oxidative DNA or RNA damage. Further studies will be needed to uncover how stress and trauma exposure may induce such chemical modifications of DNA and RNA to contribute to diverse health and disease outcomes.

Lastly, a series of review articles highlight novel roles for other signaling pathways and molecular processes. Gassen and Rein discuss the role of autophagy—an evolutionarily conserved intracellular pathway responsible for energy, organelle, and protein homeostasis—in depressive phenotypes and its potential involvement in antidepressant action. Stepan et al. discuss recent evidence suggesting a role for Hippo signaling—a signaling pathway involved in organ development, tissue homeostasis, and regeneration—in neuroplasticity and stress-related phenotypes. Gold and Kadriu provide a perspective on the physiologic and molecular mechanisms underlying the involvement of the lateral habenula—a brain region with antireward properties and bidirectional connections to the stress system—in the development of anhedonia and other depressive phenotypes. Papadopoulou et al. review how early life stressors—including those occurring before conception, *in utero*, and postnatally—get embedded to shape subsequent responses to stressful environments, and how this embedding takes place at the molecular level. By covering such diverse mechanisms and processes, these review articles underscore how several molecular and cellular mechanisms may interact at multiple levels and in complex ways to contribute to diverse outcomes after stress and trauma exposure.

In conclusion, this Research Topic has aimed at providing up-to-date evidence on the molecular mechanisms that may underlie the development of stress and trauma-related disorders. A wide range of molecular processes were examined, including inflammatory processes, circadian biology, key intracellular pathways such as autophagy and Hippo signaling, oxidative stress, and epigenetic regulation. It is our hope that this compilation and the resultant discussion will stimulate fruitful research that aims at unraveling the molecular cascade of events through which stressful experiences may contribute to the development of aberrant disease phenotypes. Furthermore, while keeping in mind that these mechanisms likely act complementarily and in complex ways to shape diverse stress-related outcomes across individuals, deeper insights into the role of individual molecular mechanisms can have important implications for identifying novel molecular targets, eventually enhancing our ability to predict, prevent, diagnose, and treat stress-related disorders.

## Author Contributions

AZ wrote the first draft of the manuscript. MS and GC critically revised the manuscript. All authors read and approved the submitted version.

## Conflict of Interest

The authors declare that the research was conducted in the absence of any commercial or financial relationships that could be construed as a potential conflict of interest.

## References

[1] Schmitt A Malchow B Hasan A Falkai P The impact of environmental factors in severe psychiatric disorders. Front Neurosci (2014) 8:19. 10.3389/fnins.2014.00019 24574956 PMC3920481

[2] Kendler KS Karkowski LM Prescott CA Causal relationship between stressful life events and the onset of major depression. Am J Psychiatry (1999) 156(6):837–41. 10.1176/ajp.156.6.837 10360120

[3] Zannas AS Jia M Hafner K Baumert J Wiechmann T Pape JC Epigenetic upregulation of FKBP5 by aging and stress contributes to NF-kappaB-driven inflammation and cardiovascular risk. Proc Natl Acad Sci U S A (2019) 116(23):11370–9. 10.1073/pnas.1816847116 PMC6561294 31113877

